# Tetraploidy-Associated Genetic Heterogeneity Confers Chemo-Radiotherapy Resistance to Colorectal Cancer Cells

**DOI:** 10.3390/cancers12051118

**Published:** 2020-04-30

**Authors:** Claudia Galofré, Öykü Gönül Geyik, Elena Asensio, Darawalee Wangsa, Daniela Hirsch, Carolina Parra, Jordi Saez, Meritxell Mollà, Zeynep Yüce, Antoni Castells, Thomas Ried, Jordi Camps

**Affiliations:** 1 Gastrointestinal and Pancreatic Oncology Team, Institut D’Investigacions Biomèdiques August Pi i Sunyer (IDIBAPS), Hospital Clínic de Barcelona, Centro de Investigación Biomédica en Red de Enfermedades Hepáticas y Digestivas (CIBEREHD), 08036 Barcelona, Spain; claudiagalofre@gmail.com (C.G.); neko.nyaa84@gmail.com (E.A.); carolina12pg@gmail.com (C.P.); CASTELLS@clinic.cat (A.C.); 2 Section for Cancer Genomics, Genetics Branch, National Cancer Institute, National Institute of Health, Bethesda, MD 20817, USA; oykugonul89@gmail.com (Ö.G.G.); wangsada@mail.nih.gov (D.W.); daniela.hirsch@nih.gov (D.H.); 3 Department of Medical Biology, Faculty of Medicine, Dokuz Eylul University, 35330 Izmir, Turkey; zeynyuce@gmail.com; 4 Radiation Oncology Department, Hospital Clínic de Barcelona, 08036 Barcelona, Spain; josaez@clinic.cat (J.S.); MOLLA@clinic.cat (M.M.); 5 Unitat de Biologia Cel·lular i Genètica Mèdica, Departament de Biologia Cel·lular, Fisiologia i Immunologia, Facultat de Medicina, Universitat Autònoma de Barcelona, 08193 Bellaterra, Spain

**Keywords:** tetraploidy, whole-genome duplication, chemotherapy, radiotherapy, drug resistance, colorectal cancer

## Abstract

Tetraploidy, or whole-genome duplication, is a common phenomenon in cancer and preludes chromosome instability, which strongly correlates with disease progression, metastasis, and treatment failure. Therefore, it is reasonable to hypothesize that tetraploidization confers multidrug resistance. Nevertheless, the contribution of whole-genome duplication to chemo-radiotherapy resistance remains unclear. Here, using isogenic diploid and near-tetraploid clones from three colorectal cancer cell lines and one non-transformed human epithelial cell line, we show a consistent growth impairment but a divergent tumorigenic potential of near-tetraploid cells. Next, we assessed the effects of first-line chemotherapeutic drugs, other commonly used agents and ionizing radiation, and found that whole-genome duplication promoted increased chemotherapy resistance and also conferred protection against irradiation. When testing the activation of apoptosis, we observed that tetraploid cells were less prone to caspase 3 activation after treatment with first-line chemotherapeutic agents. Furthermore, we found that pre-treatment with ataxia telangiectasia and Rad3 related (ATR) inhibitors, which targets response to replication stress, significantly enhanced the sensitivity of tetraploid cells to first-line chemotherapeutic agents as well as to ionizing radiation. Our findings provide further insight into how tetraploidy results in greater levels of tolerance to chemo-radiotherapeutic agents and, moreover, we show that ATR inhibitors can sensitize near-tetraploid cells to commonly used chemo-radiotherapy regimens.

## 1. Introduction

Tetraploidy, the state of having four sets of each chromosome as a consequence of whole-genome duplication (WGD), is a common feature of cancer cells [1]. Several lines of evidence indicate that WGD facilitates the acquisition of a transformed phenotype in human cancers [2], by creating an intermediate state that results in highly aneuploid unstable karyotypes [3]. A seminal study to prove the role of tetraploidy in tumorigenesis showed that p53-null tetraploid cells were able to generate tumors when injected into nude mice, whereas the diploid counterparts did not [4]. In fact, tumors that had undergone genome doubling display a higher tolerance to genomic instability resulting in tumor progression [5]. Additionally, WGD was suggested to act as a driver event that arises early in some cancers [6] and was shown to predict worse overall survival in different cancer types, independent of established clinical prognostic factors [7].

It is well-known that tetraploidy can be induced through different mechanisms, including cytokinesis failure, endoreduplication, mitotic slippage and, less commonly, cell fusion [8]. Recently, environmental stress such as hyperthermia was shown to cause polyploidization in several cancer cell lines [9]. In principle, newly generated tetraploid cells are more likely to result in highly aneuploid karyotypes across consecutive cell divisions due to increased rates of chromosome mis-segregation, which might hamper cellular growth [10]. However, tumor cells can alleviate the impaired fitness. For example, inactivation of the Hippo pathway, either by depletion of LATS2 or by expression of a constitutively active version of YAP, is sufficient to restore proliferative capacity of tetraploid cells [11]. Moreover, other studies suggested that DNA metabolism is compromised in chromosomal unstable cells [12] and near-tetraploid cells [13], which display higher amounts of replication stress than stable and diploid counterparts, respectively. Several mechanisms, by which mild replication stress might cause aneuploidy, have been described, including increased mitotic microtubule growth rates [14] and centrosome disengagement [15].

A direct consequence of tetraploidy is the increase in the levels of chromosome instability (CIN), resulting in highly aneuploid genomes [5,13]. It was suggested that CIN results in tumors with karyotypically heterogeneous subpopulations that will often outcompete other tumor cell populations when exposed to selective pressures [16,17]. Therefore, it is plausible to hypothesize that CIN and tetraploidization confer multidrug resistance, including resistance to some of the most commonly used chemotherapeutic agents, by creating greater levels of genetic diversity and thus promoting the emergence of resistant clones [18,19,20]. In fact, previous studies already suggested high sensitivity of tetraploid clones to the inhibition of cell cycle or mitotic regulators, including checkpoint kinase 1 [21], aurora kinase B [22], kinesin family member 11 [23], and MPS1 [24]. Nevertheless, there is still an unmet need to identify novel strategies to eradicate polyploid cells by identifying unique features and mechanisms to sensitize these cells to conventional chemo-radiotherapeutic treatments [25].

In the present work, we systematically assessed proliferation rates and levels of resistance to first-line and other commonly used chemotherapeutic agents as well as to ionizing radiation of tetraploid colorectal cancer (CRC) cell lines and non-transformed cells compared to their diploid counterparts. Additionally, we observed that treating tetraploid cells with an ATR inhibitor, which targets response to replication stress, reverted the resistant phenotype and decreased the number of viable cells to those levels observed in their diploid counterparts. This study provides evidence that tetraploidy is associated with multi-drug resistance, and thus novel strategies are needed to identify the Achilles’ heel of tetraploid cells to induce sensitivity to current cancer treatments.

## 2. Results

To assess to what extent tetraploidy confers therapy resistance, we used three CRC cell lines (DLD-1, RKO and SW837), the human non-transformed RPE1 cell line, and their tetraploid counterparts. DLD-1 and RKO are mismatch repair deficient colon cancer cells with pseudodiploid stable karyotypes (2N = 46 for DLD-1 and 2N = 47–50 for RKO). In contrast, SW837 is a mismatch repair proficient rectal cancer cell line with a 2N = 40 [26,27]. Near-diploid and near-tetraploid (hereinafter referred to as 2N and 4N, respectively) clones for each of the CRC cell lines were generated in our laboratory and levels of chromosome instability were previously described [13].

### 2.1. Growth Retardation of Near-Tetraploid Cells in Untreated Conditions and Diverse Tumorigenic Potential

**Figure 1:**
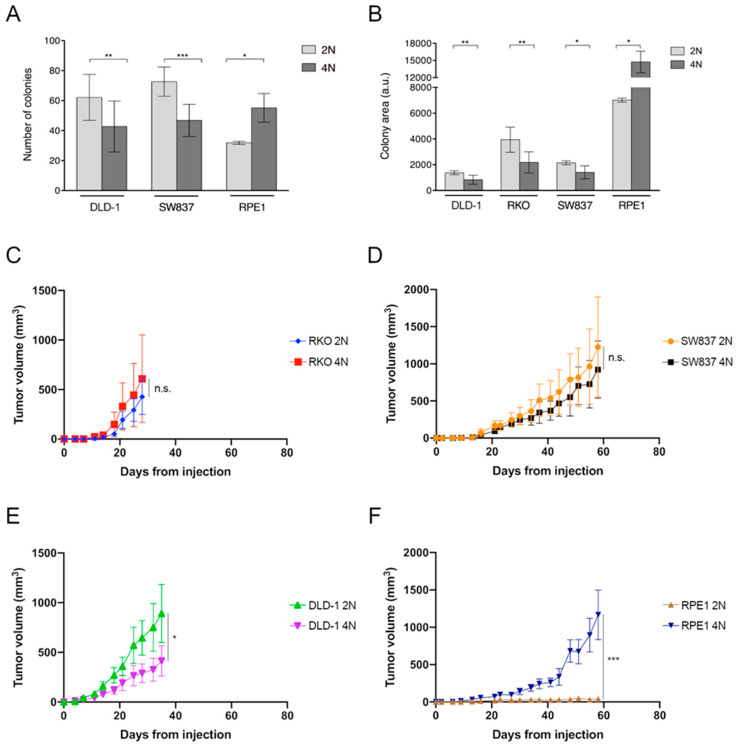
Proliferative and tumorigenic capacities of 2N and 4N cells. Quantification of colony formation assay by (**A**) number of colonies and (**B**) area of occupancy in one 2N and two 4N clones derived from the different cell lines. Please note that analysis of the number of colonies for the RKO cell line was not possible due to its dispersed phenotype in the colony-forming assay. Mean of at least two replicates from three independent experiments with SD is shown. Measurements of tumor volumes after injecting (**C**–**E**) CRC and (**F**) non-transformed RPE1 2N and 4N cells subcutaneously in athymic nude mice. A.u., arbitrary units. *, *p* < 0.05; **, *p* < 0.01; ***, *p* < 0.001.

To establish a baseline for cellular growth, we first assessed whether 2N and 4N cells exhibited the same cellular fitness in untreated conditions. Cellular proliferation analyzed by colony formation assays showed that 4N cells expanded significantly slower than 2N cells for all cancer cell lines tested, when assessing both the total number of colonies (Figure 1A and Figure S1) and the overall area of growth (Figure 1B and Figure S1). Conversely, non-transformed RPE1 4N cells displayed significantly higher proliferation capacity than 2N cells (Figure 1A,B). Of note, RPE1 cells displayed larger colony areas compared to CRC cells lines due to its growing pattern (Figure 1B and Figure S1).

To analyze their capacity to induce tumorigenesis, 1 × 10^6^ 2N and 4N cells from DLD-1, RKO, SW837 and RPE1 cell lines were injected subcutaneously in athymic nude mice. For RKO and SW837, results indicated that 4N cells show the same tumorigenic capacity as 2N cells with no statistical differences in tumor growth. As for DLD-1, 2N and 4N cells displayed the same capacity to form tumors, but 4N cells displayed a growth pattern significantly slower than 2N cells (*p* = 0.03), which is in line with the in vitro data. In contrast, tetraploidization of RPE1 cells did confer tumorigenic properties compared to the non-tumorigenic 2N RPE1 cells (*p* < 0.001) (Figure 1B–E). The higher capacity of RPE1 4N cells to induce tumorigenesis in mice agrees with their enhanced clonogenicity.

### 2.2. Near-Tetraploid Cells Exhibit Tolerance to Treatment with First-Line and Other Chemotherapeutic Agents

**Figure 2:**
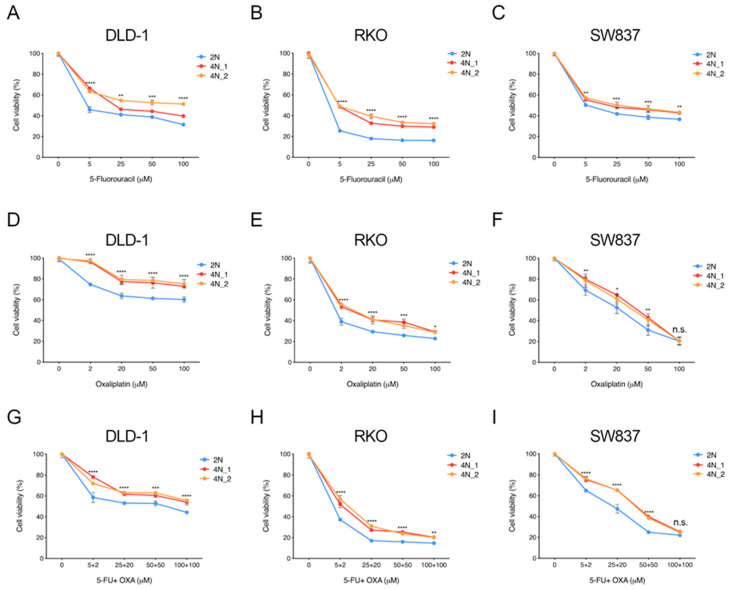
Cellular viability response upon treatment with first-line chemotherapeutic agents. Dose-response curves for increasing concentrations of 5-fluorouracil (**A**–**C**), oxaliplatin (**D**–**F**) and the combination of both compounds (**G**–**I**) in one 2N and two 4N clones of DLD-1, RKO and SW837 CRC cell lines. Each cellular viability was normalized based on its corresponding non-treated counterpart. Fitted curves for two replicates from three independent experiments are plotted. ANOVA test with post-hoc Tukey was performed to test significance. Data are reported as means ± SD. n.s., not significant; *, *p* < 0.05; **, *p* < 0.01; ***, *p* < 0.001; ****, *p* < 0.0001.

To explore to what extent tetraploidy provides a selective advantage in therapy resistance, we evaluated the effect of first-line chemotherapeutic agents used in CRC patients, i.e., 5-fluorouracil, oxaliplatin, and FOLFOX. Three CRC cell lines and the non-transformed RPE1 cells were exposed to increasing concentrations of 5-fluorouracil and oxaliplatin emulating clinically used concentrations. Cellular viability was measured at 72 hours and each experiment was performed in triplicates. Compared to its corresponding untreated cell culture, 4N clones from all cell lines showed a general multidrug resistant phenotype compared to 2N clones (Figure 2). 4N cells were significantly more resistant than their 2N counterparts to the pyrimidine analog 5-fluorouracil at a dose range from 5 to 100 µM (Figure 2A–C). Additionally, all 4N clones showed significant resistance to oxaliplatin when administrated at a dose range between 2 and 100 µM (Figure 2D–F). Similarly, post-tetraploid RPE1 also displayed increased tolerance to 5-fluorouracil and oxaliplatin to similar levels as those showed by DLD-1, but less sensitive than RKO and SW837 (Figure S2A,B). Treatment with the combination of both drugs, FOLFOX, did only show a slight additive effect in some conditions (Figure 2G–I and Figure S2C), thus suggesting that the addition of oxaliplatin on top of 5-fluorouracil did not further compromised cellular viability.

Since both 5-fluorouracil and oxaliplatin interfere with DNA synthesis, we rationalized that 4N cells might display a general resistance to these drugs due to the double amount of DNA. To test whether the increased amount of DNA in 4N clones is ultimately responsible for the tolerance to these agents, we tested 4N clones with twice the amount of drug concentration and compared with 2N clones. Even in these conditions, all 4N clones displayed higher tolerance to 5-fluorouracil (Figure S3A–C) and oxaliplatin (Figure S3D–F) treatment compared to 2N cells in all CRC cell lines. Post-tetraploid non-transformed RPE1 cells also showed a significative resistant phenotype compared to its diploid counterpart after treatment with double amount of FOLFOX (Figure S3G).

**Figure 3:**
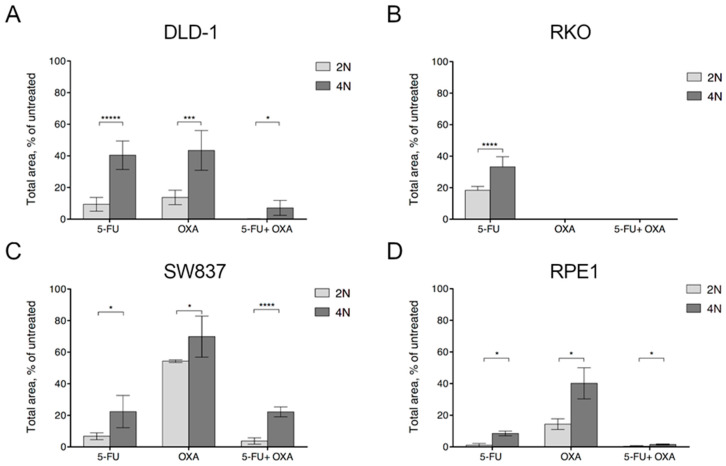
Clonogenic capacity of 2N and 4N cells after treatment with first-line chemotherapeutic agents. Graphs showing the clonogenic capacity in one 2N and two 4N clones of DLD-1 (**A**), RKO (**B**), SW837 (**C**) and RPE1 (**D**) cells treated with 5 µM 5-fluorouracil, 5 µM oxaliplatin or the combination of both compounds. Clonogenic capacity was assessed by area of occupancy over the untreated control at 14 to 21 days after treatment. Data are reported as means ± SD (*n* = 6). n.s., not significant; *, *p* < 0.05; ***, *p* < 0.001; ****, *p* < 0.0001.

To further test whether WGD promoted increased resistance to first-line chemotherapeutic agents used in CRC patients, we assessed the capacity of 2N and 4N cells to form colonies after exposure to these agents. To establish suitable doses of chemotherapy drugs, we first tested 25 µM for 5-fluorouracil and 20 µM for oxaliplatin, but no colonies were formed due to elevated toxicity. Therefore, we reduced the drug concentration to 5 µM of 5-fluorouracil and 5 µM of oxaliplatin. By assessing the area of growth using these conditions, DLD-1, RKO, SW837, and RPE1 4N cells showed higher colony formation capacity than 2N cells (Figure 3A–D). Of note, treatment with FOLFOX showed the most severe cellular viability impairment in DLD-1 cells. No colonies were observed when either 2N or 4N RKO cells were treated with oxaliplatin, suggesting that these cells are highly impaired to form colonies under this treatment (Figure 3B). In addition, not only the size but also the number of colonies was higher in 4N compared to 2N clones after treatment (Figure S4A–C). Of note, RKO cells did not form discrete colonies due to the high spread capacity, thus making it impossible to assess the readout of this assay. Overall, these results suggest that although 4N cells displayed a slower growth rate under untreated conditions, WGD promoted increased resistance to multidrug treatments.

Next, we interrogated the observed multidrug tolerance of 4N cells using other specific drugs commonly used for therapy in CRC, such as irinotecan, paclitaxel and gemcitabine. In this case, treatment was administered to DLD-1 2N and 4N clones and the MTT readout was obtained after 72 hours. Treatment with both irinotecan and gemcitabine resulted in a larger percentage of 4N cells with higher viability. Significance was reached for most of the concentrations tested for irinotecan, while only high concentrations of gemcitabine exhibited statistically significant difference (Figure S5A,B). In contrast, DLD-1 4N cells showed a statistically significant increased sensitivity to paclitaxel compared with 2N counterparts (*p* < 0.05) (Figure S5C), potentially associated with its effect on microtubule stabilization.

### 2.3. Apoptosis is Ubiquitously Activated by First-Line Chemotherapy Treatments

**Figure 4:**
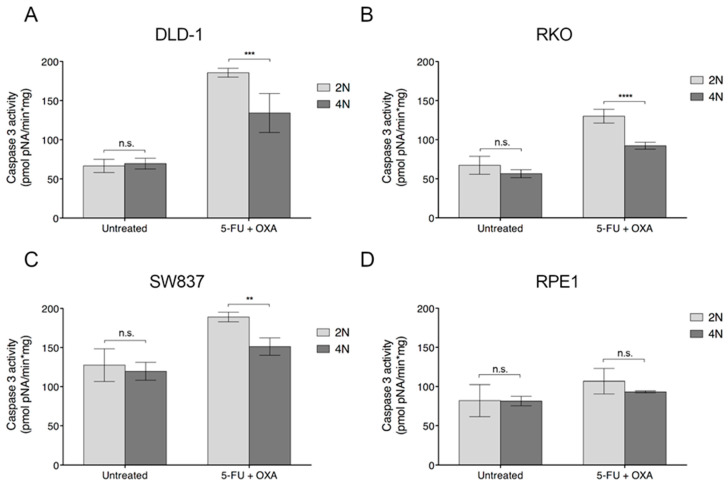
Caspase 3 activation assay in 2N and 4N cells upon treatment with FOLFOX. Quantification of caspase3 activity as a surrogate marker of apoptosis in 2N and 4N clones of (**A**) DLD-1, (**B**) RKO, (**C**) SW837 and (**D**) RPE1 cell lines in both non-treated and after treatment (25 µM of 5-fluorouracil and 20 µM of oxaliplatin) conditions. For all these analyses, caspase3 activity was calculated as pmol of pNA/min·mg of protein at 24 h after treatment. Three independent experiments were performed for each cell line. Data are represented as means ± SD. n.s., not significant; **, *p* < 0.01; ***, *p* < 0.001; ****, *p* < 0.0001.

To investigate whether the impairment in cellular viability was due to an increase in apoptosis or simply as a consequence of a reduction in cellular proliferation, we conducted a caspase 3 activity assay as a surrogate marker of apoptosis. As the activation and cleavage of caspase 3 changes over time, we first tested three different exposure times (i.e., 6, 16 and 24 h) to FOLFOX treatment (25 µM of 5-fluorouracil and 20 µM of oxaliplatin). As shown in Figure S6A, the highest caspase 3 activity was obtained at 24 hours of treatment, and thus used as a readout timepoint. Following these conditions, the caspase 3 activity assay was performed in DLD-1, RKO, SW837 and RPE1 2N and 4N cells. Results indicated that FOLFOX treatment induced apoptosis in all cell lines. Despite the fact that untreated 2N and 4N cells showed no statistically significant differences in apoptosis activity, 4N cells showed significantly less caspase 3 activation after treatment with FOLFOX for all cancer cell lines tested (Figure 4A–C). However, no differences between 2N and 4N clones were found in RPE1 cells under FOLFOX treatment (Figure 4D). To test whether caspase 3 is activated as a response to either 5-fluorouracil or oxaliplatin individually, we incubated 2N and 4N DLD-1 cells with a single drug, and confirmed that caspase 3 was already activated as a result of treating cells with individual agents (Figure S6B). Altogether these results indicate that the greater resistance of 4N cells to chemotherapy agents is, at least partially, due to the fact that 4N cells are less prone to activate apoptosis.

### 2.4. Near-Tetraploid Cells Display Resistance to Ionizing Radiation Treatment

**Figure 5:**
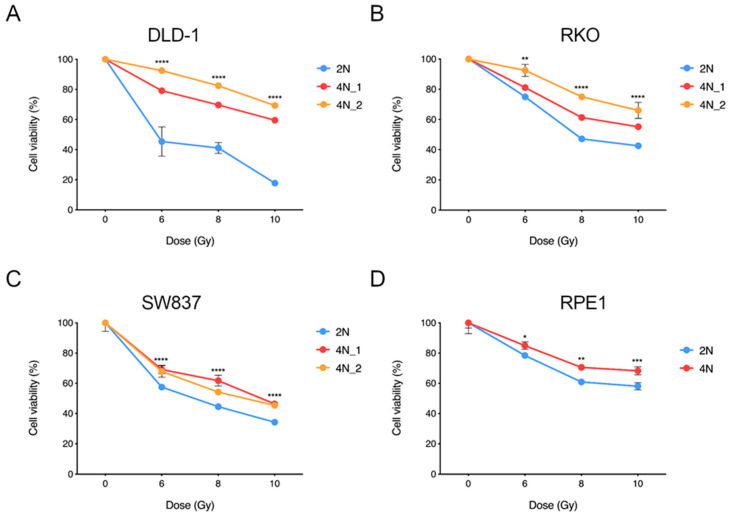
Cellular viability response upon increasing doses of ionizing radiation. Graphs depicting cellular viability curves of one 2N clone and two 4N clones of CRC cells (**A**) DLD-1, (**B**) RKO, (**C**) SW837 and (**D**) the non-transformed cell line RPE1 in response to irradiation at the indicated doses. Each cellular viability was normalized based on its corresponding non-irradiated counterpart. Fitted curves for two replicates from three independent experiments are plotted. ANOVA test with post-hoc Tukey was performed to test significance. Data are reported as means ± SD. *, *p* < 0.05; **, *p* < 0.01; ***, *p* < 0.001; ****, *p* < 0.0001.

Radiation is still the standard of care for the treatment of many cancers, including locally advanced rectal cancer. Therefore, we interrogated whether WGD was also providing resistance to ionizing radiation at similar doses as those administrated to cancer patients. To this end, we irradiated 2N and 4N clones derived from the three CRC cell lines as well as the non-transformed RPE1 cell line. A single dose of ionizing radiation at 6, 8 and 10 Gy was chosen in agreement with the Radiation Oncology Department of the Hospital Clínic of Barcelona. Cells were grown for 144 hours after irradiation and then cellular viability was assessed. Results showed that regardless of the dose, 4N cells from all four cell lines presented significant higher tolerance and greater viability compared to their 2N counterparts at the time of readout (Figure 5A–D).

### 2.5. ATR Inhibitors Sensitize Near-Tetraploid Cells to Chemo-Radiation Treatment

Finally, we hypothesized that treating 4N cells with an ATR inhibitor would sensitize these cells to treatment as they are highly dependent on the ATR dependent checkpoint kinase 1 (CHK1) due to their increased levels of replication stress. First, we confirmed that the ATR inhibitor VE-821 inhibited the ATR signaling pathway. To this end, we assessed phosphorylation of CHK1, a downstream target of ATR, by Western blot analysis after treating DLD-1 and RKO cells with 10 µM VE-821. The concentration of the ATR inhibitor was chosen based on previously reported data [13]. Our results indicated that treatment with VE-821 reduced the phosphorylation of CHK1, both in DLD-1 and RKO 2N and 4N cells (Figure S7A,B).

**Figure 6:**
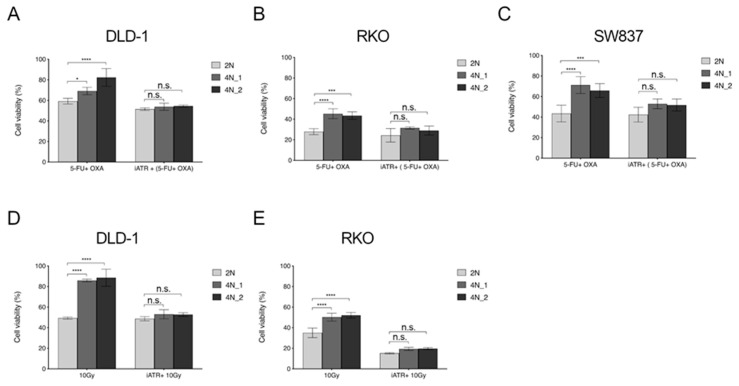
Effect of FOLFOX and ionizing radiation upon treatment of 2N and 4N cells with the ATR inhibitor VE-821. Histograms showing cell viability in response to VE-821 treatment prior to FOLFOX treatment (**A**–**C**) or ionizing radiation (**D**,**E**). One 2N and two 4N clones of (**A**) DLD-1, (**B**) RKO and (**C**) SW837 were treated with FOLFOX (25 µM 5-fluorouracil and 20 µM oxaliplatin) for 72 h, when indicated 10 µM VE-821 was added for 24 h prior to FOLFOX treatment. Same clones of (**D**) DLD-1 and (**E**) RKO were irradiated with a dose of 10 Gy, 10 µM VE-821 was added for 24 h before irradiation when indicated. Each cellular viability was normalized based on its corresponding non-treated or non-irradiated counterpart. Mean of at least three independent experiments with SD is shown. n.s., not significant; *, *p* < 0.05; ***, *p* < 0.001; ****, *p* < 0.0001.

We then tested whether VE-821 would increase the cytotoxic effects of FOLFOX by exposing 2N and 4N clones derived from DLD-1, RKO and SW837 to 10 µM of VE-821 for 24 hours before treating them with FOLFOX (25 µM of 5-fluorouracil and 20 µM of oxaliplatin) for 72 hours. Cellular viability was assessed for all three cancer cell lines and showed that VE-821 sensitizes 4N cells to FOLFOX treatment to the extent that 4N cells became as sensitive to FOLFOX as 2N cells (Figure 6A–C). Moreover, when testing the effect of combining FOLFOX treatment in VE-821 treated RPE1 cells, we observed a chemo-sensitizing effect as well (Figure S8). Likewise, the same rationale was considered when treating cells with ionizing radiation. Therefore, DLD-1 and RKO 2N and 4N cells were irradiated with a single dose at 10 Gy after being incubated with 10 µM VE-821 for 24 hours. Readout for cellular viability was obtained after one week. Results showed that inhibition of ATR radio-sensitized 4N cells to reach the same level of viability impairment than their 2N counterparts (Figure 6D,E). Taken together, pre-treatment with ATR inhibitor can reverse the chemo-radiotherapy resistant phenotype of 4N cells, achieving a sensitivity close to that of 2N cells.

## 3. Discussion

This study sheds light into how WGD and the associated levels of increased aneuploidy confer cells with increased tolerance to chemo-radiotherapeutic agents. Here, we treated 2N and 4N isogenic clones from four cell lines with an array of drugs and radiation, and showed that cellular fitness in 4N cells was higher than in the 2N counterparts. Interestingly, incubating 4N cells with an ATR inhibitor before drug treatment reversed this effect.

Several lines of evidence already suggested that pseudo-tetraploid karyotypes resulting from WGD events confer resistance to certain drug treatments. In this sense, a recent report using the CRC cell line HCT116 and derived post-tetraploid clones identified multidrug resistance associated with post-tetraploid but not diploid clones [20]. In our study, we used three CRC cell lines with distinct genetic background. Specifically, DLD-1 and RKO are mismatch repair deficient with diploid karyotypes while SW837 is a hypodiploid, CIN positive, mismatch repair proficient cell line. In contrast to HCT116, which is *TP53* wild-type, DLD-1 and SW837 are *TP53* mutated, while RKO is a *TP53* wild-type but *BRAF* mutated [28,29]. First-line chemotherapeutic treatment of advanced CRC is based on the administration of individual regimens of 5-fluorouracil or oxaliplatin or the combination of both also known as FOLFOX. Our study presents for the first time a systematic comparison of 2N and 4N clones after treatment with conventional drugs typically administered as first-time therapy to patients with CRC. An overall increase in resistance to these drugs is observed for 4N cells. Nevertheless, treatment with FOLFOX do not show an additive effect when measuring the cellular viability response; however, it does for clonogenic capacity and apoptosis activation assays. This could be due to the lower sensitivity of the cellular viability readout when using the MTS cell proliferation assay kit. Moreover, 4N cells also showed increased tolerance to irinotecan and gemcitabine, but not to paclitaxel. In contrast to irinotecan and gemcitabine (and also to 5-fluorouracil and oxaliplatin), paclitaxel is a cytoskeletal drug that promotes microtubule stabilization, cell cycle arrest and thus activates apoptosis. The fact that 4N cells are sensitive to paclitaxel might be related to the increased number of chromosomes in these cells. Of note, our data suggest that 4N cancer cells, but not the non-transformed RPE1 cells, are less sensitive to activate apoptosis when treated with chemotherapeutic drugs. The lack of significant differences in RPE1 cells could be either because the peak concentration of caspase 3 in RPE1 cells is at a different timepoint or because RPE1 cells activate some other caspase. Although it was previously shown that tetraploid cells exhibit an enhanced rate of spontaneous apoptosis in the presence of wild-type p53 [30], apoptosis resistance was linked to loss of ploidy control in Burkitt’s lymphoma after treating with nocodazole or paclitaxel [31]. Nevertheless, different sensitivities are observed across cell lines. To note, while RKO 4N displayed tolerance to 5-fluorouracil, higher sensitivity to oxaliplatin compared to DLD-1 and SW837 was observed when attempting to form colonies. These differences are intriguing since BRAF-like CRC cells display greater sensitivity to drugs affecting microtubule dynamics as well [32]. Whether cellular pathways leading to evasion of apoptosis are more frequently altered in polyploid than diploid cells remains unsolved.

Furthermore, we sought to interrogate whether the overall increase in resistance of 4N cells to chemotherapeutic agents was also seen when treating cells with ionizing radiation. Neoadjuvant radiotherapy is the standard of care to treat locally advanced rectal cancer patients [33]. Consistent with our findings, 4N cells showed higher tolerance to ionizing radiation in a 6 to 10 Gy range. Several cellular processes were implicated in resistance to irradiation treatments. Specifically, in CRC cells, WNT activity was previously associated with ionizing radiation resistance [34,35]. More recently, internalization of epidermal growth factor receptor (EGFR) by Rab5C was suggested to enhance resistance to ionizing radiation in rectal cancer [36]. In a different context, tetraploid cells induced by cell fusion hybrids display great capacity to nuclear reprogramming as a consequence of activating the WNT/β-catenin signaling pathway [37]. Whether isogenic 4N cells have higher amounts of WNT/β-catenin activity compared to their counterparts is of utmost interest for future studies.

One significant consequence of WGD events is the presence of CIN and aneuploidy. Increased levels of CIN were previously related to multidrug resistance in several cancers [19]. One possible explanation is the fact that the higher the CIN the greater the amount of subclonal cellular populations and genetic heterogeneity [38]. Nevertheless, our results show that 4N cancer cells tend to proliferate slower than their counterparts. This is in agreement with previous reports assessing the effects of single-chromosome trisomic derivates, which showed that aneuploidy could compromise the proliferation capacity [10]. The slower proliferative rates in 4N cells might reflect a sub-population with a quasi-quiescent cell resistant to the anti-proliferative chemo-radiotherapeutic agents, a phenomenon known as infrequent cell cycle [25], and previously associated with the c-Yes/YAP Axis [39]. Of note, when assessing the tumorigenic potential of 4N clones, only the non-transformed RPE1 cells clearly benefit from duplicating the genome to increase the capacity to form tumors in nude mice. Such tetraploid-induced tumorigenicity acquisition of RPE1 cells is in agreement with the results of the clonogenic assay and with previous assessment of anchorage-independent growth [20].

Another explanation to understand why near-tetraploid cells are more tolerant to chemotherapeutic agents than diploid cells could be that WGD may buffer genome insults induced by chemo-radiotherapeutic agents [40]. Recently, it was shown that WGD might facilitate the rapid acquisition of genomic alterations and is responsible for improving the cellular fitness as a consequence of mitigating the presence of deleterious mutations and homozygous deletions [41]. Actually, in diploid yeast genetic backgrounds, it was shown that mutator phenotypes outcompete against non-mutator haploids [42]. Additionally, one cannot discard the possibility that 4N cells are less prone to succumb to chemo-radiotherapeutic agents due to the low rate of drug per nucleotide, especially for intercalating agents. However, our data showed that even when treating 4N cells with double the amount of 5-fluorouracil or oxaliplatin, the resistant phenotype persists. Considering that WGD events were suggested for numerous human cancers [1] and, according to the data presented here, these would promote treatment resilience, it is not surprising that genome doubling is associated with poor prognosis [7].

The identification of subclonal populations containing increased ploidy contents within a tumor would allow eliminating the most aggressive cancer cells. Several studies pursued the idea of treating tumors to sensitize such aggressive subclonal populations. Therefore, identifying the Achilles’ heel of tetraploid cells might eventually improve treatment. Previous reports already postulated that WGD increases tumor sensitivity through the inhibition of several cellular components mostly related to DNA damage and mitotic regulators [25]. Of note, inhibition of CHK1 was suggested to eradicate tetraploid tumor cells through a p53-dependent pathway [21]. Given the fact that tetraploid cells show increased levels of replication stress compared to their diploid counterparts [13], inhibiting ATR, the DNA damage mediated response effector to replication stress, sensitizes tetraploid cells to chemotherapeutic drugs and ionizing radiation, impairing cellular fitness to the same extent observed in diploid cells.

In summary, we conclude that WGD and its associated CIN phenotype provides resistance to the standard of care first-line and other chemotherapeutic agents as well as to ionizing radiation often used in CRC patients. In addition to the fact that WGD is associated with a protective effect of tetraploid cells against drug treatment, our data demonstrate that tetraploid cells are highly sensitive to inhibition of DNA replication stress mediating response proteins, suggesting a therapeutic strategy to sensitize them to chemo-radiotherapeutic agents.

## 4. Materials and Methods 

### 4.1. Cell Lines and Reagents

DLD-1, RKO and SW837 cells were obtained from the American Type Culture Collection (Manassas, VA, USA). DLD-1 and SW837 cells were cultured in RPMI 1640 medium and RKO cells were cultured in DMEM/F-12 medium (Life Technologies, Carlsbad, CA, USA), both supplemented with antibiotics and 10% fetal bovine serum (FBS) (Life Technologies) at 37 °C in 5% CO_2_.

Near-diploid and near-tetraploid clones of DLD-1, RKO and SW837 cell lines were previously generated in the lab [13]. Wild-type and post-tetraploid clones derived from the hTERT-immortalized retinal-pigmented epithelial cells (RPE1) (kindly provided by Z. Storchova, University of Kaiserslautern, Kaiserslautern, Germany) were cultured in DMEM/F-12 medium supplemented with antibiotics and 10% FBS at 37 °C in 5% CO_2_. For the experiments described in this study, we used one 2N clone and two 4N clones derived from the DLD-1, RKO and SW837 cell lines for reproducibility. For the RPE1 cell line, we used one 2N and one 4N clones.

Oxaliplatin (Selleckchem, Houston, TX, USA) was dissolved in dH_2_O in 5 mM aliquots. 5-fluorouracil (Sigma-Aldrich, St. Louis, MO, USA) was dissolved in dimethyl sulfoxide (DMSO) and stored in 384 mM aliquots. The range of drug concentrations for 5-fluorouracil and for oxaliplatin was 5–100 µM and 2–100 µM, respectively. The ATR inhibitor VE-821 (Selleckchem) was also dissolved in DMSO in 10 mM aliquots. Irinotecan (Sigma-Aldrich) and paclitaxel (Sigma-Aldrich) were dissolved in DMSO in 80 mM and 10 mM aliquots, respectively. Gemcitabine (Sigma-Aldrich) was dissolved in water to make 33.3 mM aliquots. Drug ranges for irinotecan, paclitaxel and gemcitabine were 0.5–20 µM, 1–100 nM and 10–1000 nM, respectively.

### 4.2. Irradiation

In vitro irradiation experiments were performed using a Gulmay 200 KV X-ray unit at a dose rate of 1.4 Gy/min at room temperature in the Radiotherapy Oncology Service of the Hospital Clinic of Barcelona. To do this, 2000 cells were seeded in 96-well plates and incubated for 24 h before exposing them to ionizing radiation with a single dose of 6, 8 or 10 Gy.

### 4.3. Cell Viability Assay

To test chemotherapeutic response, cells were plated in 96-well plates at 3000 cells per well. After 24 h, cells were treated with increasing concentrations of oxaliplatin, 5-fluorouracil or both components, and plates were further incubated for another 72 h before viability was measured. For the radiation experiments, cell viability was measured 144 h post-irradiation. When required, cells were treated with 10 µM of the ATR inhibitor VE-821 for 24 h before adding a combination of 5-fluorouracil and oxaliplatin (FOLFOX) or ionizing radiation. Viability was measured using the CellTiter96 AQueous One Solution Cell Proliferation Assay, also known as MTS (Promega, Madison, WI, USA). Absorbance was measured using the Epoch microplate reader (BioTek Instruments, Winooski, VT, USA). Each cellular viability was normalized to its corresponding non-treated counterpart. The MTT Cell Proliferation assay (Sigma-Aldrich) was used to assess viability after treatment with irinotecan, gemcitabine, and paclitaxel drugs. Absorbance was measured by SpectraMax M2 and analyzed using the software SoftMax Pro (Molecular Devices, Sunnyvale, CA, USA).

### 4.4. Colony Formation

To assess for colony formation in both treated and untreated conditions, 100 cells of each clone were plated in triplicate in a 6-well plate. After a 24 h period, cells were treated with 5 µM of oxaliplatin, 5 µM of 5-fluorouracil or the combination of both drugs. Drug washout was performed 72 h post-treatment. Cells were then incubated for 12–14 days and stained with 0.5% crystal violet (Sigma-Aldrich). Colonies were both manually counted and imaged on the EliSpot plate reader (Autoimmun diagnostika GmbH, Strassberg, Germany). Digital images were quantified by ImageJ (Image Processing and Analysis in Java; National Institutes of Health, Bethesda, MD, USA; http://imagej.nih.gov/).

### 4.5. Apoptosis Assay

Apoptosis was assessed by measuring the caspase 3 activity using a colorimetric caspase-3 activity assay (Sigma-Aldrich). Briefly, DLD-1 2N and 4N cells were treated with 20 µM of oxaliplatin, 25 µM of 5-fluorouracil or FOLFOX. RKO, SW837 and RPE1 cell lines were treated with FOLFOX. After 24 h, cells were lysed in lysis buffer and protein concentrations were determined by using the Pierce BCA Protein Assay Kit (Thermo Fisher, Waltham, MA, USA). Same amounts of each protein extract (100 µg) were assayed with the colorimetric caspase-3 substrate Ac-DEVD-pNA. The release of the yellow chromophore p-nitroanilide (pNA) was measured in an Epoch Microplate Spectrophotometer (BioTek) at 405 nM. Caspase-3 activity was calculated in comparison to a pNA standard curve.

### 4.6. Lysate Preparation and Western Blot Analysis

Cells were exposed to 10 µM VE-821 for 24 h before lysis in RIPA buffer (50 mM Tris-HCl, 1% NP-40, 0.5% Na-deoxycholate, 0.1% SDS, 150 mM NaCl, 2 mM EDTA, and 50 mM NaF, with protease and phosphatase inhibitors). Protein samples were resolved in 4% to 12% SDS-PAGE gels and electroblotted onto a PVDF membrane. The membrane was blocked in 5% milk and tris-buffered saline, 0.1% Tween-20 (TBST) for 1 h, incubated with primary antibody diluted in blocking solution overnight at 4°C, washed 3 times with TBST, incubated with secondary antibodies for 1 h at room temperature, and washed 3 times with TBST. For the detection of signals, SuperSignal West Femto (Thermo Fisher Scientific) was used. Membranes were imaged on an Image LAS400 device (GE Healthcare Life Sciences, Chicago, IL, USA).

Primary antibodies and corresponding dilutions used are as follow: rabbit anti–phospho-CHK1 (1:500; Cell Signaling Technology, Danvers, MA, USA) and mouse anti-GAPDH (1:1000; Invitrogen, Carlsbad, CA, USA). Blots were detected using goat anti- rabbit and goat anti-mouse (1:2500, Thermo Fisher Scientific). ImageJ was used for signal quantification.

### 4.7. Tumorigenic *in vivo* Assay

Athymic nude mice were used to perform subcutaneous xenografts. To do so, 1 × 10^6^ cells from one 2N and one 4N clone of each cell line were injected subcutaneously in each flank of athymic NCr-nu/nu (nude) mice with 200 μL of matrigel (1:1 mixture of PBS and growth factor reduced matrigel, BD Biosciences, San Jose, CA, USA ). The total number of replicates were 4 for RPE1 and SW837 and 5 for DLD-1 and RKO. All mice were bred and housed in a pathogen-free environment and used in experiments in accordance with institutional guidelines at the Center for Cancer Research, National Cancer Institute, National Institutes of Health. All experimental procedures conducted in this study were approved by the Animal Care and Use Committee (National Institutes of Health, the Animal Study Protocol number is MB045 and the latest approval was on 11 January 2019). Tumor sizes were measured in two dimensions twice a week, and volumes were calculated using the formula for a rotational ellipsoid v = π/6 × a × b^2^ [43]. Mice were sacrificed due to tumor size (maximum 2 cm in either length or width) 28 or 35 days after injection, respectively. For RPE1, monitoring was extended to 58 days.

### 4.8. Statistical Analysis

Statistical analysis was performed using the software Prism v8 (GraphPad Software, San Diego, CA, USA), and appropriate tests are indicated in the figure legends for each analysis when different from Student’s *t*-test. Differences in tumorigenicity in vivo between 2N and 4N clones were assessed by two-way ANOVA.

## 5. Conclusions

Tetraploidy, or whole-genome duplication, is a frequent event in tumors of epithelial origin. However, the cellular and physiological consequences of these events are still undetermined. It is well established that tetraploid cells display increased CIN, which is associated with poor prognosis. Here, we show that tetraploidy-induced CIN is able to generate a broad range of subclonal populations and provides the tumor with higher chances of becoming refractory to first-line and other chemotherapeutic treatments, probably due to a poor efficacy to activate apoptosis. In addition, this therapy resilience is also observed when tetraploid cells are treated with ionizing radiation. Intriguingly, we showed that incubating tetraploid cells with an ATR inhibitor, cell viability was reduced to the levels observed in their diploid counterparts, suggesting a synergistic effect of the ATR inhibitor and chemo-radiotherapeutic agents. This dual-treatment will need to be reproduced in primary CRC samples to validate its potential clinical impact.

## Supplementary Materials

The following are available online at https://www.mdpi.com/2072-6694/12/5/1118/s1, Figure S1: Examples of the clonogenic capacity in untreated DLD-1 and RPE1 2N and 4N cells compared with treatment with 5-fluorouracil, oxaliplatin and FOLFOX; Figure S2: Cellular viability after treating wild-type and post-tetraploid RPE1 cells with 5-fluorouracil, oxaliplatin and the combination of both; Figure S3: Cellular viability after treating cells with double the amount of chemotherapeutic agents; Figure S4: Capacity to form colonies of 2N and 4N cells after treatment with first-line chemotherapeutic agents; Figure S5: Treatment of DLD-1 2N and 4N cells with the agents irinotecan, gemcitabine and paclitaxel; Figure S6: Time-course analysis of caspase 3 activity; Figure S7: Densitometry analysis and immunoblots of cells treated with the ATR inhibitor VE-821; Figure S8: Viability assessment after treatment with the ATR inhibitor VE-821 in RPE1 cells.
