# Supplementary material for: Tetraploidy-Associated Genetic Heterogeneity Confers Chemo-Radiotherapy Resistance to Colorectal Cancer Cells
Source: Cancers (Basel). 2020 Apr 30;12(5):1118. doi: 10.3390/cancers12051118 (PMC7281619; doi:10.3390/cancers12051118)
Supplement: Supplementary file 1 [file cancers-12-01118-s001.pdf]

Supplementary Files

Tetraploidy-Associated Genetic Heterogeneity Confers Chemo-Radiotherapy Resistance to Colorectal Cancer Cells

Claudia Galofré, Öykü Gönül Geyik, Elena Asensio, Darawalee Wangsa, Daniela Hirsch, Carolina Parra, Jordi Saez, Meritxell Mollà, Zeynep Yüce, Antoni Castells, Thomas Ried and Jordi Camps

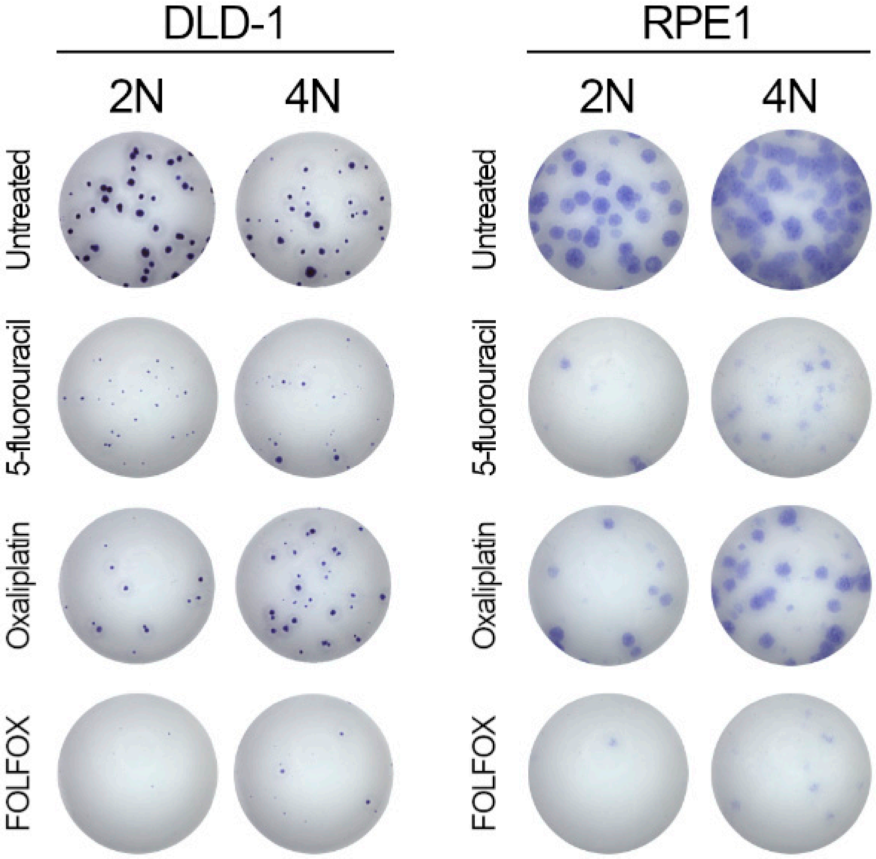

Figure S1. Examples of the clonogenic capacity in untreated DLD-1 and RPE1 2N and 4N cells compared with treatment with 5-fluorouracil, oxaliplatin and FOLFOX. Note the difference in the colony area between DLD-1 and RPE1 cells.

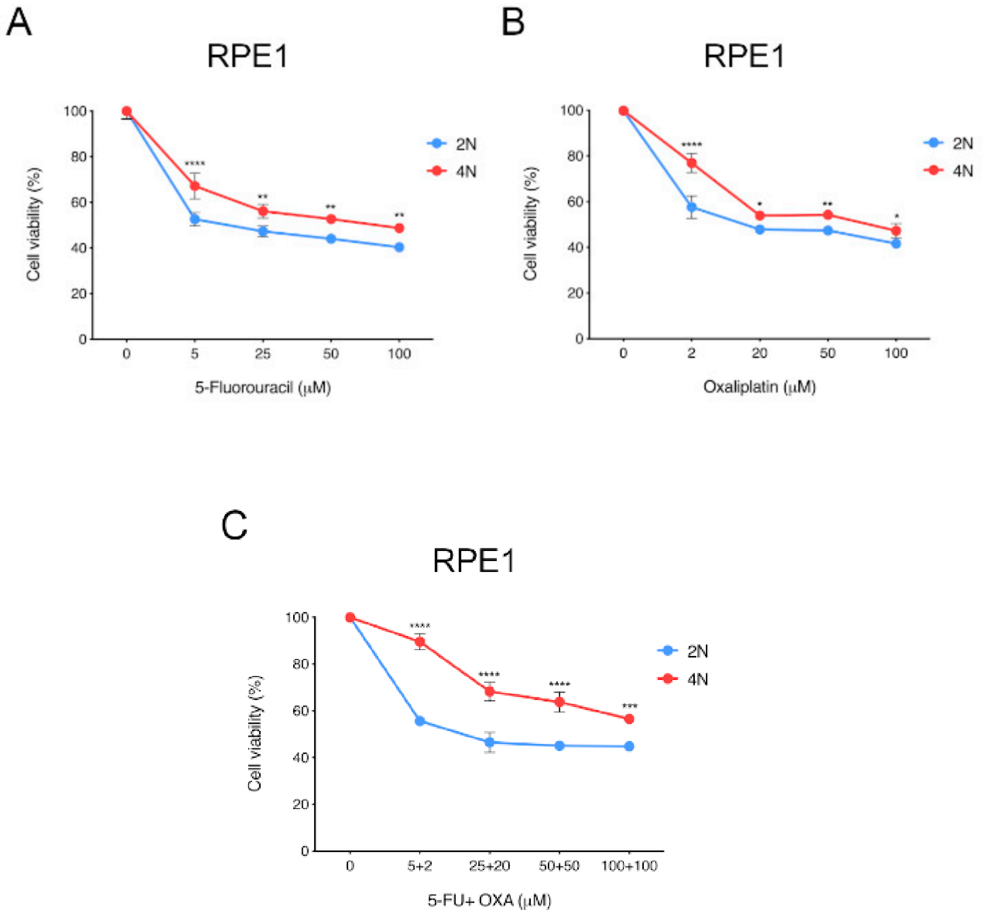

Figure S2. Cellular viability after treating wild-type and post-tetraploid RPE1 cells with 5-fluorouracil, oxaliplatin and the combination of both. Dose-response curves showing cellular viability for 2N and 4N clones of RPE1 after treatment with scaling concentrations of (**A**) 5-fluorouracil, (**B**) oxaliplatin or (**C**) combination of both. Each cellular viability was normalized based on its corresponding non-treated counterpart. Plotted is fitted curve for two replicates from three independent experiments. ANOVA test with post-hoc Tukey was performed to test significance. Data are reported as means ± SD. *, $p < 0.05$; **, $p < 0.01$; ***, $p < 0.001$; ****, $p < 0.0001$.

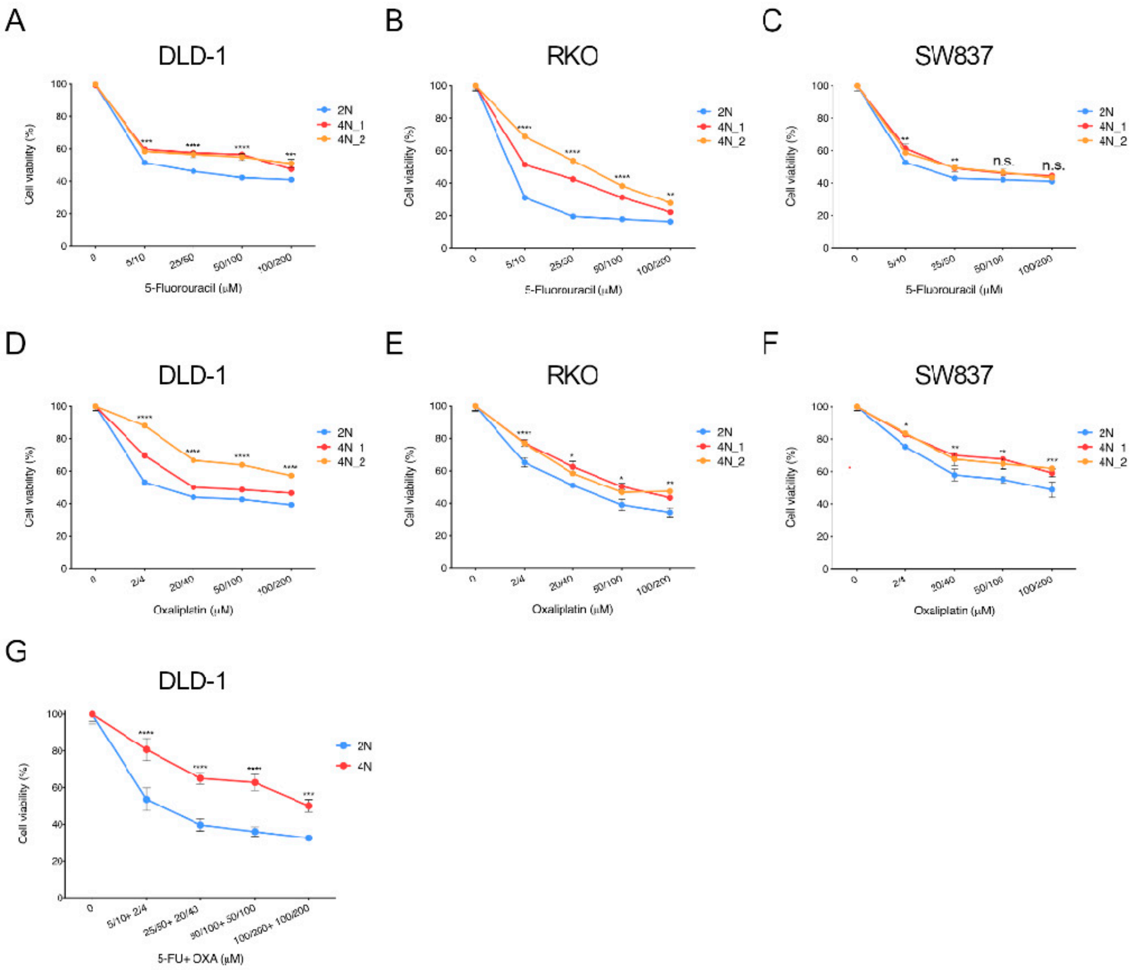

Figure S3. Cellular viability after treating cells with double the amount of chemotherapeutic agents. Dose-response curves depicting cellular viability after treatment with increasing concentrations of 5-fluorouracil (**A–C**) or oxaliplatin (**D–F**) for one 2N and two 4N clones of DLD-1 (**A,D**), RKO (**B,E**) and SW837 (**C,F**) cell lines. (**G**) RPE1 cell line clones were treated with scaling doses of combination of both agents. Note that twice the amount of chemotherapeutic agents was used for these analysis. Each cellular viability was normalized based on its corresponding non-treated counterpart. Fitted curve for two replicates from three independent experiments is plotted. To test significance ANOVA test with post-hoc Tukey was performed. Data are reported as means ± SD. n.s., not significant; *, $p < 0.05$; **, $p < 0.01$; ***, $p < 0.001$; ****, $p < 0.0001$.

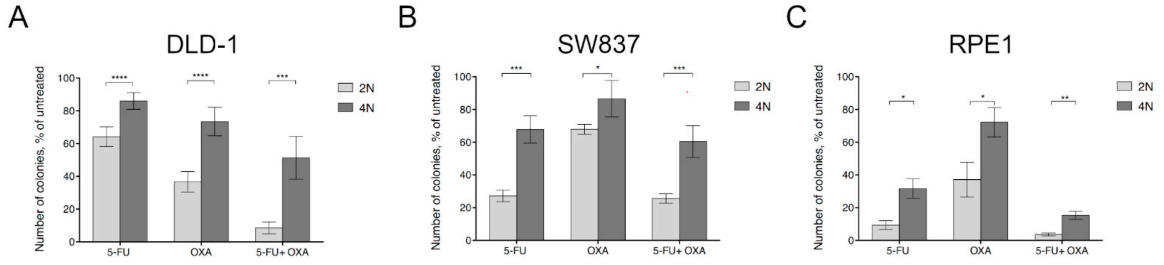

Figure S4. Capacity to form colonies of 2N and 4N cells after treatment with first-line chemotherapeutic agents. Quantification of clonogenic capacity for one 2N and two 4N clones of (**A**) DLD-1 and (**B**) SW837 CRC cell lines, and (**C**) one 2N and one 4N clones of RPE1 non-transformed cell line was assessed after treatment with 5 μM of 5-fluorouracil, 5 μM of oxaliplatin or combination of both agents. Clonogenic capacity was evaluated by counting the number of colonies grown over the untreated control at 14 to 21 days after treatment. Analysis of RKO cell line was not possible due to colony-forming dispersed phenotype. Data are reported as means ± SD (n = 6). n.s., not significant; *, $p < 0.05$; **, $p < 0.01$; ***, $p < 0.001$; ****, $p < 0.0001$.

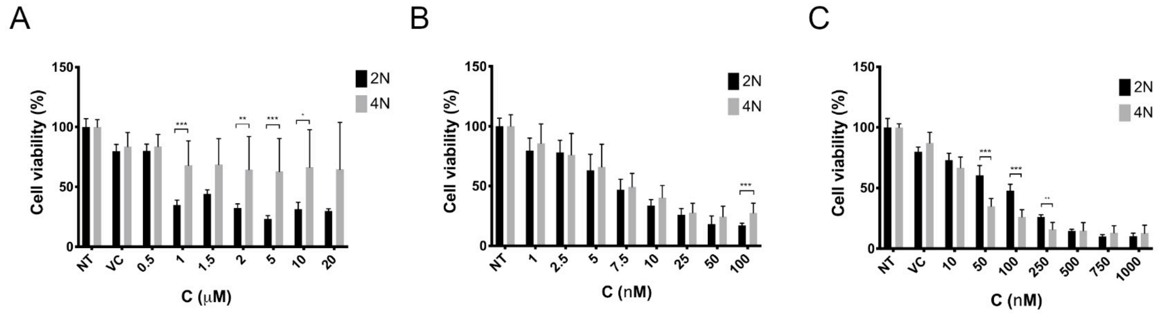

Figure S5. Treatment of DLD-1 2N and 4N cells with the targeted agents (**A**) irinotecan, (**B**) gemcitabine, and (**C**) paclitaxel. Viability was measured by the MTT Cell Proliferation assay. Data are reported as means ± SD ($n = 3$). *, $p < 0.05$; **, $p < 0.01$; ***, $p < 0.001$.

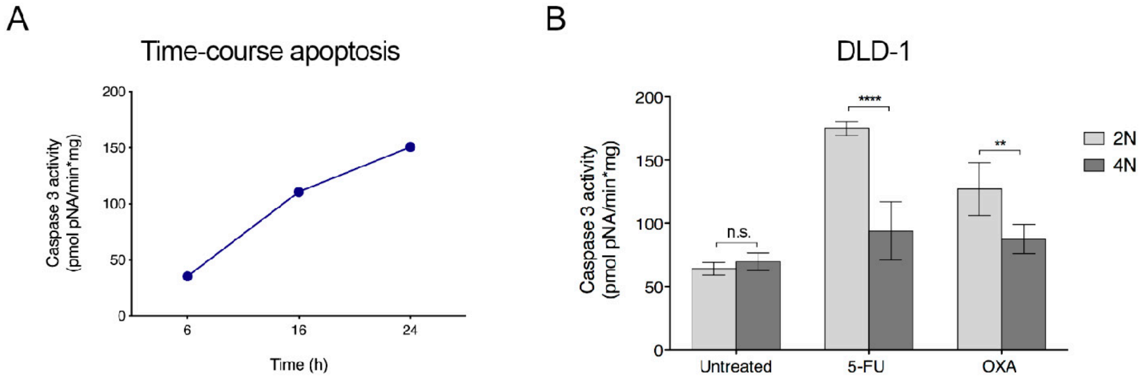

Figure S6. Analysis of caspase 3 activity. (**A**) Assessment of caspase 3 activity at different time-points after chemotherapeutic treatment. Caspase3 activity was calculated as pmol of pNA/min·mg of protein at 6, 16 and 24 h after treatment. (**B**) Graph depicting caspase 3 activity of non- treated 2N and 4N DLD-1 cells and after treating them with 25 μM 5-fluorouracil or 20 μM oxaliplatin. Caspase3 activity was calculated as pmol of pNA/min·mg of protein at 24 h after treatment. Three independent experiments were performed for each treatment. Levels represented as means ± SD. n.s., not significant; **, $p < 0.01$; ****, $p < 0.0001$.

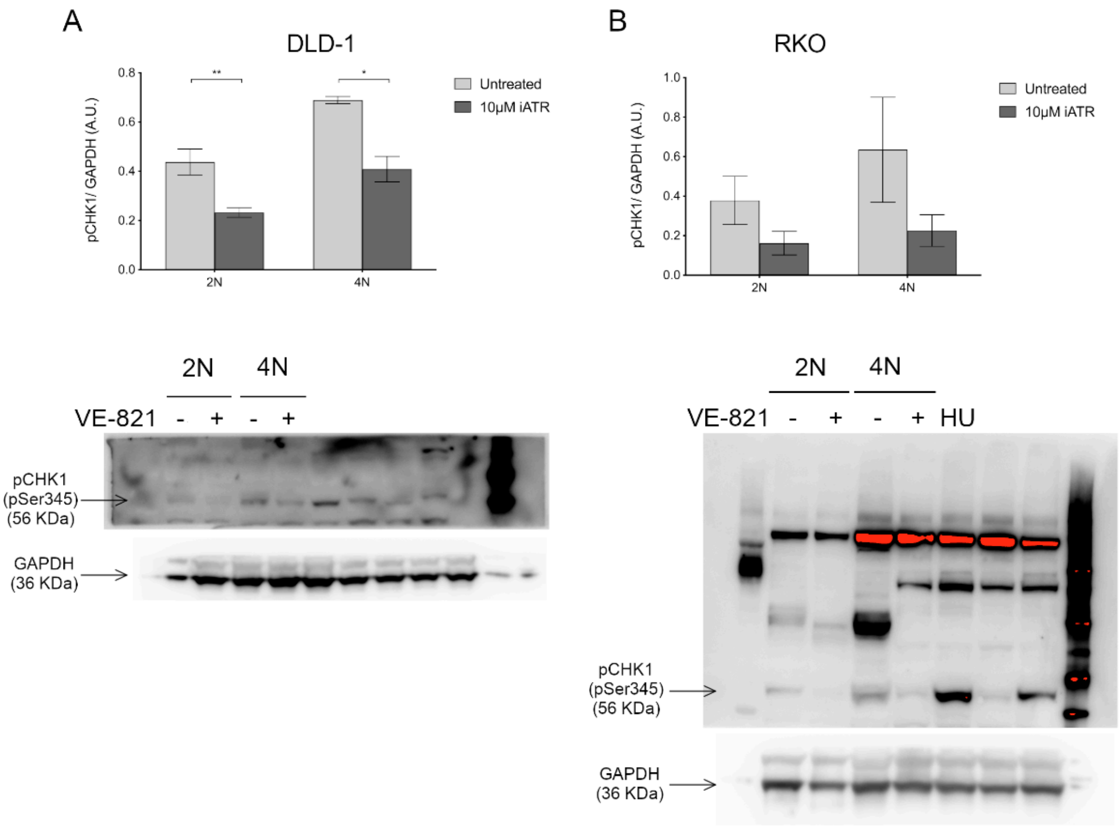

Figure S7. Densitometry analysis and immunoblots of cells treated with the ATR inhibitor VE-821. (**A**) Densitometry analysis of DLD-1 cell line and the corresponding full length Western-Blot of pCHK1 (pSer345) and the loading control GAPDH from different parts of the same gel. Mean of 3 independent experiments with SD is shown. *, p < 0.05; **, p < 0.01. (**B**) Densitometry analysis of RKO cell line and the corresponding full length Western-Blot of pCHK1 (pSer345) and the loading control GAPDH from different parts of the same gel. Hydroxyurea (HU) was used in this experiment as positive control. Mean of 2 independent experiments with SD is shown.

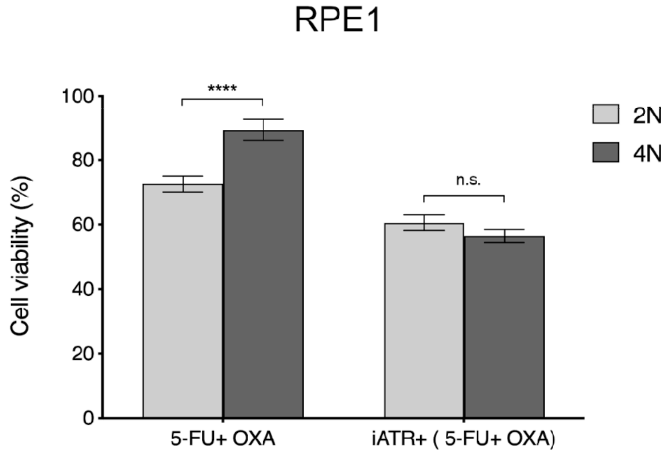

Figure S8. Viability assessment after treatment with the ATR inhibitor VE-821 in RPE1 cells. Histogram depicting cellular viability of RPE1 2N and 4N cells treated with VE-821 and first-line chemotherapeutic agents. Platted cells were treated with 25 μM 5-fluorouracil and 20 μM oxaliplatin, when indicated 10 μM VE-821was added for 24 h prior to treatment. Each cellular viability was normalized over non-treated control. Mean of at least 3 independent experiments with SD is shown. n.s., not significant; ****, $p < 0.0001$.

© 2020 by the authors. Licensee MDPI, Basel, Switzerland. This article is an open access article distributed under the terms and conditions of the Creative Commons Attribution (CC BY) license (http://creativecommons.org/licenses/by/4.0/).
